# COVID-19—Related Assault on Asians: Economic Hardship in the United States and India Predicts Diminished Support for Victim Compensation and Assailant Punishment

**DOI:** 10.3390/ijerph18105320

**Published:** 2021-05-17

**Authors:** James Johnson, David N. Sattler, Kylie Otton

**Affiliations:** 1 Department of Social Sciences, Laucala Campus, The University of the South Pacific, Suva 1168, Fiji; johnsonj2323@gmail.com; 2 Department of Psychology, Western Washington University, Bellingham, WA 98225-9172, USA; ottonk@wwu.edu

**Keywords:** Asian assault, Asian discrimination, economic hardship, coronavirus, COVID-19, pandemic, coronavirus pandemic, violence, discrimination, assault

## Abstract

*Background*: There has been an alarming increase in discrimination and violence towards Asians during the coronavirus (COVID-19) pandemic amid reports that the virus was first detected in China. In an incident involving a COVID-19-related physical assault, this study examined whether economic hardship experienced by participants during the pandemic and the race of the victim (Chinese, White) would influence support to compensate a victim and punish the assailant. The study also explored whether the perception that the victim experienced emotional and physical suffering due to the assault would mediate the relationships. *Method*: Participants in India and the United States reported on their own economic hardship due to the pandemic. They then read about an incident in which an innocent person suffered a COVID-19-related physical and verbal attack, and indicated if they would support punishing the assailant and financially compensating the victim. *Results*: When the victim was Chinese, participants experiencing a high degree of COVID-19 economic hardship were less likely to support financially compensating the victim or punishing the assailant compared to when the victim was White. Furthermore, when the victim was Chinese, the negative associations between economic hardship and financially compensating the victim and punishing the assailant were mediated by reduced recognition that the victim suffered emotional trauma and pain as a result of the attack. *Conclusions*: COVID-19-driven economic hardship experienced by participants predicted an array of reactions that reflected reduced recognition of the civil and human rights of a victim of a COVID-19-related assault. These findings have significant implications for mental health, public health, and the justice system, and underscore the pressing need for prompt action to mitigate economic hardship and to address racism and discrimination.

## 1. Introduction

There has been an alarming increase in discrimination and violence towards Asians during the coronavirus (COVID-19) pandemic amid reports that the virus was first detected in China [1,2]. In a review of anti-Asian discrimination incidents during the pandemic, the Asian Pacific Policy and Planning Council reports that witnesses and bystanders oftentimes did not intervene or provide any form of support to the victim [3,4]. For example, in a pharmacy in the United States, an Asian woman was attacked when another customer covered her in Lysol disinfectant spray and yelled that she was “the infection.” Although others observed the incident, “no one came to help her” [4]. In New York City, observers did not intervene or provide assistance when a man repeatedly kicked a Filipino woman in the chest and yelled vulgarities at her [5]. In India, customers in a restaurant joined others in harassing an Asian woman and forced her to leave [6]. In Germany, an Asian woman was harassed on a train and was stunned when no one in the car assisted her and, days after the incident, when she had ”not seen or heard any German politician or major influencer coming to our defense” [3]. For her, the lack of response from the observers—their silence and indifference—was as troubling as the violent act itself. During the pandemic, the World Health Organization, American Public Health Association, American Medical Association, American Psychological Association, and other public health entities worldwide have firmly reiterated their position that racism is a serious public health crisis that demands immediate attention.

This study examined how economic hardship due to the COVID-19 pandemic influences social complicity in response to assaults on Asians [7,8]. Examining responses to Asian mistreatment is critically important [9]. First, prompt action is needed to address the public health crisis posed by the disturbing rise in violence and discrimination. Second, societal complicity (e.g., through apathy, indifference, and failure to intervene to assist a victim) may lead to a normalization of hate crimes, wherein perpetrators may believe they can engage in violence “boldly and with impunity” [10] (p. 3). Third, the public’s understanding and interpretation of criminal behavior as well as the responses of the justice system to criminal actions have profound implications for victims, perpetrators, and public health in general [11]. Fourth, citizens who serve as jurors in court cases are typically required to render important decisions that carry substantial consequence, such as determining whether to formally charge, convict, and/or punish an individual accused of the crime, and whether to compensate the victim.

This study addresses three pressing issues: Will economic hardship during the COVID-19 pandemic influence support to criminally charge an individual who instigates a COVID-19-related assault on an Asian victim? Will economic hardship predict whether individuals support financially compensating a victim of a COVID-19-related assault? Will perceptions concerning the degree to which the victim suffered mediate the association between economic hardship and support for punishing the assailant, and support for compensating the victim?

### 1.1. Economic Deprivation and Minority Group Bias: New Applications and Directions

Financial stress as a result of the economic downturn during the COVID-19 pandemic may be responsible, in part, for aggression directed at Asians [7,8]. Research shows that economic hardship is associated with an increase in negative attitudes towards minorities. For example, Bianchi and colleagues showed that during an economic downturn, Whites reported feeling less warmly and held increased negative explicit and implicit attitudes towards Blacks. Further, they were more likely to condone the use of stereotypes and to regard inequality as natural and acceptable [12]. Other studies show that during economic downturns, Black musicians were less likely to secure a musical hit, and Black politicians were less likely to win a congressional election. Further, the perception that resources are scarce is associated with an increase in negative beliefs and altered perceptions of Black faces in a manner that facilitate discrimination [13,14] and the exclusion of individuals who are biracial [15]. While research examining economic distress and prejudice towards minorities has tended to focus on general attitudes and perceptions [14,16] and allocation of resources [17], there is a lacuna of research examining factors that influence judgments people make about physical violence against minorities, including support for the perpetrator and the victim.

In their seminal work examining economic depression and violence toward minorities in the Southern United States between 1882 and 1930, Hovland and Sears reported that a decline in economic indicators was associated with Black lynchings by Whites [18]. However, despite the evidence that economic distress is associated with greater violence against minorities, there has been surprisingly little direct empirical examination of how economic distress will influence reactions to such violence. Notably, some social scientists contend that the aversive nature of discriminatory violent actions may create a climate of social intolerance, wherein individuals would express their condemnation by supporting legal sanctions for the perpetrator, confronting the perpetrator, and publicly denouncing the violent acts [19]. The present study extends the economic stress and prejudice literature by focusing on reactions to violence. First, we examine whether economic stress will predict support to compensate the victim (i.e., compensatory reactions). Second, we examine whether economic stress will predict support to punish the individual accused of the crime (i.e., punitive reactions towards the assailant). Third, we explore whether the perception that the victim experienced emotional and physical suffering will mediate the relationship of economic stress with compensatory reactions and punitive reactions.

### 1.2. A Motivated Perception Perspective

To provide a bit of theoretical leverage, Krosch and Amodio have proposed a motivated perception perspective to explain why economic hardship might lead to greater prejudice and bias towards minority group members [13]. The basic tenet is that economic scarcity motivates majority group members to engage in perceptual processes that can disadvantage a minority group in various ways. The perspective is based in classic prejudice research demonstrating that intergroup economic competition can lead individuals to perceive outgroup members as more threatening which, in turn, facilitates greater prejudice and discrimination [20,21]. In a series of four studies, Krosch and Amodio show that compared to participants in the control condition, those assigned to an economic scarcity condition reported that they perceived Black people as “darker” and as “more stereotypically Black” [13]. Further, participants experiencing this perceptual change allocated fewer resources to Black recipients compared to those in the control condition. In a later study, Krosch and Amodio reported another consequence of economic scarcity: it can disrupt the encoding of minority group faces which, in turn, can facilitate discrimination (viz., the allocation of fewer resources to Blacks) [22]. There is also evidence that the perception of pain and suffering experienced by others is associated with motivational processes. For example, unwillingness to acknowledge suffering experienced by disliked others or to take steps to relieve their pain is associated with activity in the “reward” centers of the brain [23,24]. Further, counter-empathic responding (i.e., schadenfreude) tends to increase when a disliked-other suffers a significant injury [25].

### 1.3. Overview and Predictions

The central goal of this study was to examine how economic hardship experienced during the pandemic (e.g., loss of job, food insecurity) and the race of a victim a COVID-19-related physical assault (Chinese, White) would influence reactions to the assault. Given that the pandemic has resulted in economic hardship and has adversely affected public health worldwide [26,27], we were particularly interested in examining these questions in countries where persons who are Chinese are a minority. As such, we conducted this study in India and the United States; both countries have extensive coronavirus spread and are experiencing significant economic hardship due to the pandemic.

Participants were randomly assigned to read a passage describing a White male’s unprovoked physical attack on either a Chinese or White male due to his belief that the victim had COVID-19. Participants then reported the degree to which (a) the victim experienced emotional trauma, (b) the victim experienced physical pain, (c) they supported financially compensating the victim (i.e., victim compensation support), and (d) they endorsed criminally charging the assailant (i.e., perpetrator-directed punitive reactions). We expected an interaction between economic hardship and victim race such that the negative association between economic hardship and perceived emotional trauma (Hp1), perceived physical pain (Hp2), victim compensation support (Hp3), and perpetrator-directed punitive reactions (Hp4) would be greater for the Chinese victim relative to the White victim. We further expected that moderated-mediational analysis would reveal that for the Chinese victim (but not the White victim) (a) reduced perceived emotional trauma (Hp5) and reduced perceived physical pain (Hp6) would mediate the expected negative association between economic hardship and endorsement of financially compensating the victim, and (b) reduced perceived emotional trauma (Hp7) and reduced perceived physical pain (Hp8) would mediate the expected negative association between economic hardship and perpetrator-directed punitive reactions.

While the assessment of the participant country was exploratory (thus no specific predictions), there is evidence that persons in India and the United States tend to have similar views about COVID-19 in relation to its country of origin [28,29,30,31]. Consequently, individuals from both countries may be susceptible to similar perceptual and evaluative consequences due to COVID-19 being first detected in China [6]. Thus, this study considers whether exposure to similar stereotypical information will facilitate “cross-cultural” similarities in violence-related reactions towards minorities [32].

## 2. Method

### 2.1. Participants, Materials, and Procedure

The participants were 202 persons in the United States (120 male, 81 female, 1 not reported) and 203 persons in India (153 male, 50 female) (for a total of 405 participants) who were recruited using the crowdsourcing website Amazon Mechanical Turk (MTurk). The race of participants in the United States was as follows: White (*N* = 170), Black (*N* = 18), and Latinx (*N* = 14). The average age was 36.72 (*SD* = 11.03, range 18–78), and most (87%) had some college or a college degree. The race of participants in India was Indian (*N* = 203). The average age was 29.37 (*SD* = 5.20, range 21–49), and most (99%) had some college or a college degree.

Participants responded to a study titled “Decision Making” and received financial compensation for their participation. To ensure the quality of the data, participants had to report a MTurk reputation score of 0.90 or greater [33]. The sample size was based on the following factors: (a) it is similar to or far exceeds what was used in past research on reactions to minority-directed physical violence [32], (b) the power analysis revealed that the number was more than sufficient, and (c) it was within our budget. The research was approved and conducted in compliance with the host institution’s Internal Review Board.

Participants were presented with informed consent information, and if consent was given, they first completed five items assessing their own personal COVID-19–related economic hardship using a 7-point scale (1 = strongly disagree to 7 = strongly agree, α = 0.89). Sample items are “I have lost income from my job as a result of the coronavirus,” and “I am concerned that I am unemployed now or will be in the coming months because of the coronavirus situation.” In order to isolate the impact of general stress from economic hardship, participants completed the Perceived Stress Scale using a 5-point scale (1 = never to 5 = often; α = 0.77) [34]. Sample items are “In the last month, how often have you felt nervous and stressed?” and “In the last month, how often have you been angered because of things that were outside of your control?”.

Participants were then informed that the researchers were interested in how individuals make decisions about various life events. They read a “newspaper story” involving a White male who, while on a train, approached either a Chinese male or a White male fellow passenger who was wearing a face mask. Participants were randomly assigned to condition. The newspaper story presented the race of all parties involved in the incident. The story revealed that the White male assailant shouted loudly to the victim, “Why are you wearing the mask—you must be carrying the coronavirus! I saw you sneeze earlier so you shouldn’t be out!” The victim calmly replied that he only sneezed because he has allergies. The assailant continued to make disparaging statements and subsequently kicked the victim in the chest three times, which knocked him to the floor. He then stood over the victim and said, “You had better get off at the next stop because you are putting us all in danger! I do not want to get sick!”. At that point, other passengers subdued the assailant and contacted the police, who arrested him at the next stop.

After reading the passage, participants completed several measures, using a 5-point scale (1 = not at all to 5 = very much) to indicate their responses. Perceived victim emotional trauma was assessed by an item that measured the extent to which participants were certain that the victim experienced emotional trauma from the assault. Perceived victim physical pain was assessed by an item that measured the extent to which participants were certain that the victim experienced physical pain from the assault. Victim compensation support was assessed by participants indicating the extent that, if they were actual jurors, they were certain that they would support the victim receiving financial compensation if he sued the perpetrator. Finally, perpetrator-directed punitive reactions were assessed by an item focused on the extent to which participants felt that, if they were actual jurors, they would support the perpetrator being criminally charged (i.e., indicted) for his actions. Previous research shows that these or similar items accurately measure responses to vignettes describing an incident of interracial violence [32].

Debriefing statements included a discussion of the importance of cultural diversity, intergroup respect, nonviolence, etc.

### 2.2. Statistical Analysis Plan

#### 2.2.1. Analysis of the Hypothesized Interaction

Personal COVID-19-related economic hardship is a continuous variable, and PROCESS Model 3 [35] was run to test the significance of the victim race (Chinese, White) x participant country (United States, India) x economic hardship interaction. PROCESS Model 1 was utilized to test the more central victim race x reported economic hardship interaction expectations. PROCESS [35] is a SPSS [36] macro that executes path analysis-based moderation and mediation analysis using bootstrapping measures with 10,000 sample.

#### 2.2.2. Probing the Interactions

The PROCESS Macro Model 1 provides a method to examine the expected differences in the association between reported economic hardship and the relevant outcome measures in the Chinese versus White victim conditions.

#### 2.2.3. Moderated-Mediation

PROCESS Model 7 [35] was run to test the expected mediation of the association between reported economic hardship and the relevant outcome measures (i.e., victim compensation, perpetrator-directed punitive reactions) by emotional trauma and physical pain perceptions in the Chinese (but not the White) victim condition.

## 3. Results

### 3.1. Power Analysis

Our sampling strategy was informed following prospective power analyses for ordinary least squares (OLS) linear regressions and independent t-tests (corrected for multiple comparisons) respectively. A prospective analysis for an OLS regression producing a moderate effect (f^2^ = 0.15) with 99% power recommended a sample size of n = 164 (GPower) [37]. A prospective analysis for a two-sided test producing a moderate effect (.3) with 99% power suggested a sample of *N* = 247. Our final sample of *N* = 405 was appropriate for highlighting small-to-moderate effects with high power across all analyses reported.

### 3.2. Preliminary Analysis: Establishing the Independence of Economic Hardship from Victim Race

The overall effect of victim race on participant-reported economic hardship did not reach significance, *t*(403) = 0.48, *p* = 0.631, η_2_ = 0.001. Thus, it can be concluded that random assignment eliminated any systematic difference between the conditions in terms of economic distress.

A significant effect for participant country, *t*(403) = 10.98, *p* < 0.00, η_2_ = 0.230, revealed that participants in India (*M* = 3.49, *SD* = 0.72) reported greater economic hardship than those in the United States (*M* = 2.43, *SD* = 1.16). However, this was not relevant for our purposes because participant country did not moderate the interaction of victim race and reported economic hardship for any of the relevant outcome variables. Further, the victim race x economic hardship interaction remained significant for all outcome variables after controlling for participant country.

For the entire results section, all variability in degrees of freedom is due to missing values.

### 3.3. Correlations between Major Variables

Table 1 presents the correlation matrix broken down by victim race.

### 3.4. Perceived Victim Emotional Trauma

There was no effect of victim race on perceived victim emotional trauma, *t*(403) = 1.92, *p* = 0.054, η^2^ = 0.009. Relative to participants in India (*M* = 3.68, *SD* = 1.03), participants in the United States (*M* = 3.91, *SD* = 0.99) reported greater perceived victim emotional trauma, *t*(403) = 2.24, *p* = 0.025, η^2^ = 0.012.

**Figure 1:**
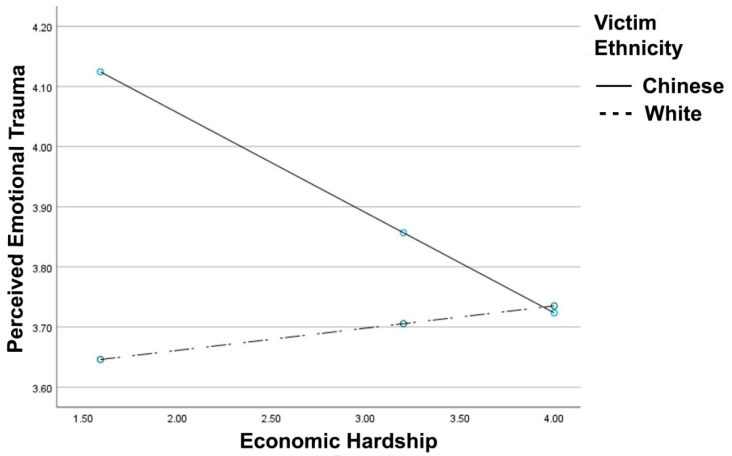
Perceived emotional trauma as a function of reported economic hardship and victim ethnicity. Greater values indicate greater perceived emotional trauma and economic hardship.

Consistent with Hypothesis 1 (see Figure 1), victim race x economic hardship interaction for perceived victim emotional trauma reached significance, *R*^2^ *Change* = 0.012, *F*(1, 401) = 4.98, *p* = 0.026, B = 0.20, 95% CI [0.02, 0.38]. For the Chinese victim, greater economic hardship was associated with reduced sensitivity to the victim’s emotional trauma, B = −0.16, *t* = −2.50, *p* = 0.012. For the White victim, there was no association between economic hardship and perceived victim trauma, B = 0.03, *t* = 0.59, *p* = 0.552. Further, the victim type x economic hardship x participant country interaction did not reach significance (*p* = 0.085). Finally, the interaction remained significant after controlling for age, education, and stress, *R*^2^ *Change* = 0.015, *F*(1, 398) = 6.22, *p* = 0.013, B = 0.22, 95% CI [0.04, 0.39].

### 3.5. Perceived Victim Pain

The effect of victim race on perceived victim pain did not reach significance, *t*(403) = 0.31, *p* = 0.754, η^2^ = 0.000. The effect of participant country on perceived pain did reach significance, *t*(403) = 3.40, *p* < 0.001, η^2^ = 0.028. Participants in the United States (*M* = 3.99, *SD* = 1.04) perceived greater victim pain than participants in India (*M* = 3.65, *SD* = 0.97).

**Figure 2:**
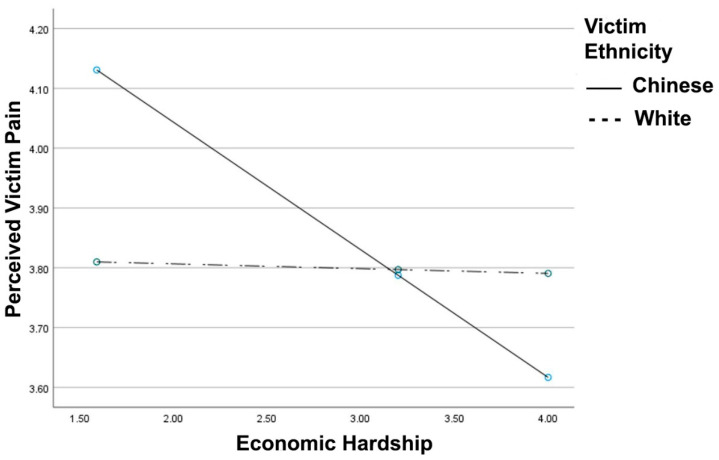
Perceived victim pain as a function of reported economic hardship and victim ethnicity. Greater values indicate greater perceived victim pain and economic hardship.

Consistent with hypothesis 2 (see Figure 2), the victim race x economic hardship interaction for perceived victim pain reached significance, *R*^2^ *Change* = 0.012, *F*(1, 401) = 5.08, *p* = 0.024, B = 0.20, 95% CI [0.03, 0.38]. For the Chinese victim, greater economic hardship was associated with reduced sensitivity to the victim’s pain, B = −0.21, *t* = −3.20, *p* = 0.001. For the White victim, there was no association between economic hardship and perceived pain, B = −0.008, *t* = −0.12, *p* = 0.898. Further, the victim type x economic hardship x participant country interaction did not reach significance for perceived pain (*p* = 0.618).

To fully isolate the impact of reported economic hardship, we included an analysis of age, education, and general stress as covariates for all interactions. The victim race x economic hardship interaction for perceived victim pain remained significant after controlling for age, education, and stress, *R*^2^ *Change* = 0.013, *F*(1, 398) = 5.22, *p* = 0.022, B = 0.20, 95% CI [0.03, 0.38].

### 3.6. Victim Compensation Support

The effect for victim race on victim compensation support reached significance, *t*(403) = 2.21, *p* = 0.027, η^2^ = 0.012. The Chinese victim (*M* = 3.76, *SD* = 1.06) received greater support than the White victim (*M* = 3.52, *SD* = 1.12). The effect for participant country did not reach significance, *t*(403) = 1.25, *p* = 0.212, η^2^ = 0.004.

**Figure 3:**
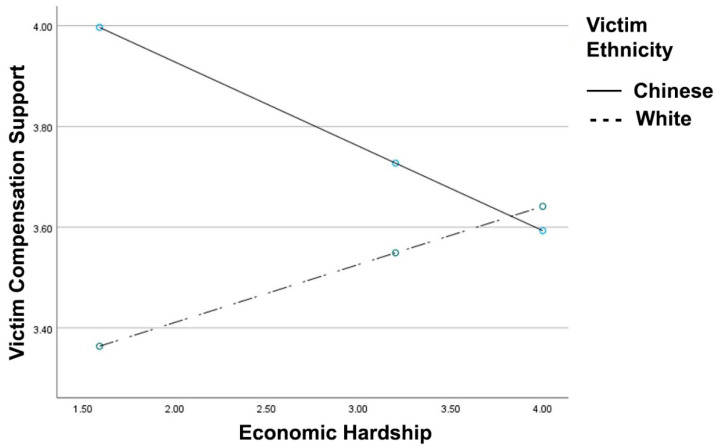
Victim compensation support as a function of reported economic hardship and victim ethnicity. Greater values indicate greater support to compensate the victim and economic hardship.

In support of Hypothesis 3 (see Figure 3), the victim race x economic hardship interaction for victim compensation support reached significance, *R*^2^ *Change* = 0.020, *F*(1, 398) = 8.31, *p* = 0.004, B = 0.28, 95% CI [0.09, 0.47]. For the Chinese victim, greater economic hardship was associated with reduced support for compensation, B = −0.16, *t* = −2.33, *p* = 0.019. For the White victim, economic hardship was not associated with compensation support, B = 0.11, *t* = 1.72, *p* = 0.086. Further, the victim type x economic hardship x participant country interaction did not reach significance for compensation support (*p* = 0.877). Finally, the victim race x economic hardship interaction remained significant after controlling for age, education, and stress, *R*^2^ *Change* = 0.020, *F*(1, 398) = 8.85, *p* = 0.003, B = 0.28, 95% CI [0.09, 0.47].

### 3.7. Support to Criminally Charge the Assailant (Perpetrator-Directed Punitive Reactions)

The effect of victim race did not reach significance for perpetrator-directed punitive reactions, *t*(403) = 0.33, *p* = 0.735, η^2^ = 0.000, or participant country, *t*(403) = 1.47, *p* = 0.142, η^2^ = 0.005.

**Figure 4:**
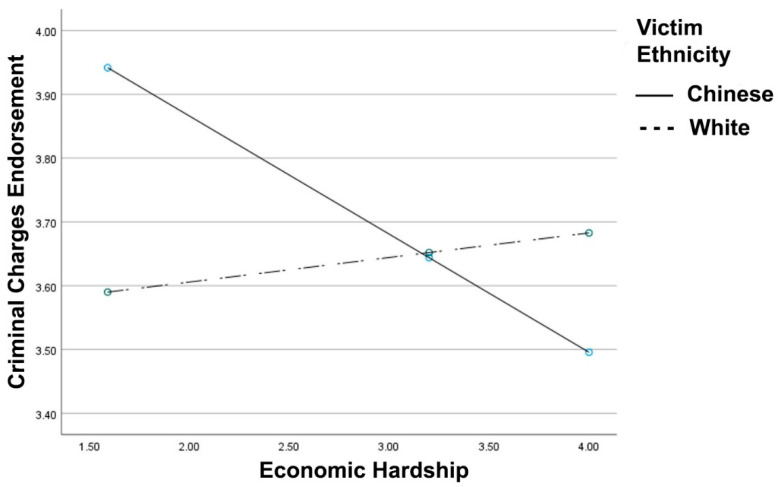
Support to criminally charge the perpetrator (criminal charges endorsement) as a function of reported economic hardship and victim ethnicity. Greater values indicate greater support for criminal charges and economic hardship.

Consistent with hypothesis 4 (see Figure 4), the victim race x economic hardship interaction for perpetrator-directed punitive reactions reached significance, *R*^2^ *Change* = 0.011, *F*(1, 401) = 4.50, *p* = 0.034, B = 0.22, 95% CI [0.02, 0.43]. In the Chinese victim condition, greater economic hardship was associated with reduced punitive reactions for the assailant, B = −0.18, *t* = −2.40, *p* = 0.016. In the White victim condition, economic hardship was not associated with punitive reactions, B = 0.03, *t* = 0.53, *p* = 0.593. The victim type x economic hardship x participant country interaction did not reach significance for charges endorsement (*p* = 0.988).

Finally, the victim race x economic hardship interaction remained significant after controlling for age, education, and stress, *R*^2^ *Change* = 0.012, *F*(1, 398) = 5.28, *p* = 0.022, B = 0.23, 95% CI [0.03, 0.44].

### 3.8. Mediational Effects

**Figure 5:**
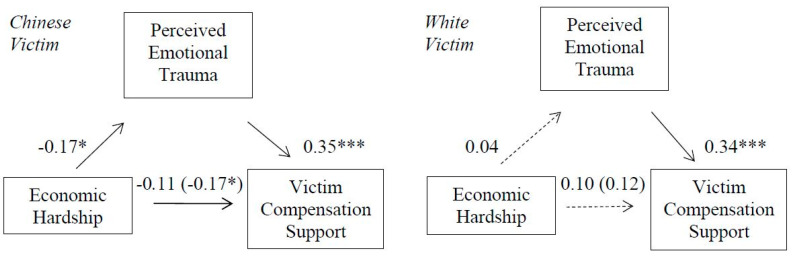
Indirect effect of economic hardship on victim compensation support through perceived emotional trauma for Chinese (**left**) and White (**right**) victim. Non-significant direct effects indicated by dashed lines. Direct effects of economic hardship on compensation support are shown in parenthesis. * *p* < 0.05. *** *p* < 0.001.

In support of hypothesis 5 (see Figure 5), a PROCESS Macro Model 7 [35] significant *moderated-mediation* effect demonstrated that perceived victim emotional trauma mediated the association between economic hardship and victim compensation support in the Chinese victim condition; 95% CI [−0.1009, −0.0199] but not for the White victim condition; 95% CI [−0.0326, 0.0562]. The index of moderated mediation was also significant, b = 0.07, [0.0094, 0.1379].

**Figure 6:**
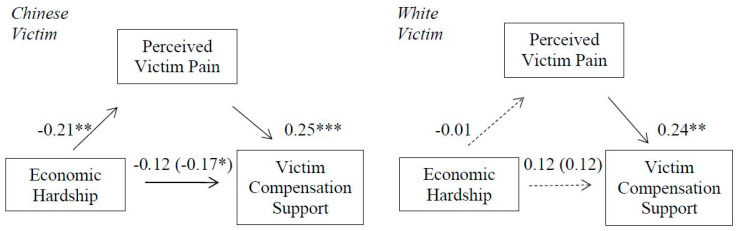
Indirect effect of economic hardship on victim compensation support through perceived victim pain perceptions for Chinese victim (**left**) and White victim (**right**). Non-significant direct effects indicated by dashed lines. Direct effects of economic hardship on compensation support are shown in parenthesis. * *p* < 0.05. ** *p* < 0.01. *** *p* < 0.001.

Hypothesis 6 was supported (see Figure 6). Perceived victim physical pain mediated the association between economic hardship and victim compensation support in the Chinese victim condition; 95% CI [−0.1116, −0.0182] but not for the White victim condition; 95% CI [−0.0320, 0.0330]. The index of moderated mediation was also significant, b = 0.05, [0.0062, 0.1136].

**Figure 7:**
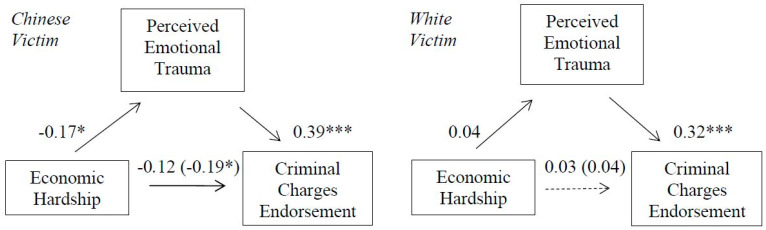
Indirect effect of economic hardship on criminal charges endorsement (punitive reactions) through perceived emotional trauma for Chinese (**left**) and White (**right**) victim. Non-significant direct effects indicated by dashed lines. Direct effects of economic hardship on criminal charges support are shown in parenthesis. * *p* < 0.05. *** *p* < 0.001.

In support of hypothesis 7 (see Figure 7), perceived victim emotional trauma mediated the association between economic hardship and endorsement for perpetrator-directed punitive responding in the Chinese victim condition; 95% CI [−0.1112, −0.0142] but not for the White victim condition; 95% CI [−0.0297, 0.0612]. The index of moderated mediation was also significant, b = 0.07, [0.0093, 0.1460].

**Figure 8:**
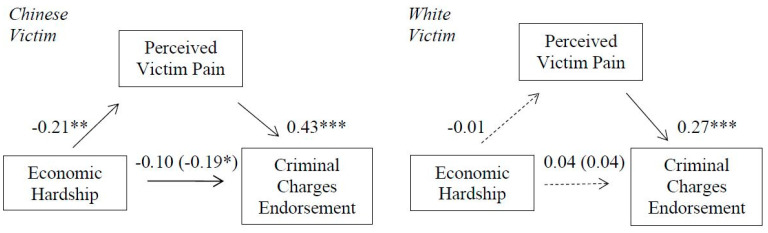
Indirect effect of economic hardship on criminal charges endorsement (punitive reactions) through perceived victim pain for Chinese (**left**) and White (**right**) victim. Non-significant direct effects indicated by dashed lines. Direct effects of economic hardship on criminal charges support are shown in parenthesis. * *p* < 0.05. ** *p* < 0.01. *** *p* < 0.001.

In support of hypothesis 8 (see Figure 8), perceived victim physical pain mediated the association between economic hardship and perpetrator-directed punitive reactions in the Chinese victim condition; 95% CI [−0.1286, −0.0272] but not for the White victim condition; 95% CI [−0.0450, 0.0454]. The index of moderated mediation was also significant, b = 0.07, [0.0080, 0.1494].

## 4. Discussion

The adverse financial and public health impact of the COVID-19 pandemic has significantly increased personal economic stress worldwide [7,27,38,39]. Amid pervasive reports that the virus originated in China, it is alarming that the pandemic has been associated with an escalation of negative and violent reactions towards Asians [40,41]. In both India and the United States, the present findings revealed bias against the Chinese victim on a variety of measures. Specifically, the *negative associations* between economic hardship experienced by participants and the perception that the victim experienced emotional trauma, the perception that the victim experienced physical pain, support to compensate the victim, and support to punish the assailant were greater when the victim was Chinese relative to the White victim. Moreover, the moderated-mediational analyses revealed that when the victim was Chinese (but not White), the *negative associations* of economic hardship with support to compensate the victim and support to punish the assailant were mediated by the perception that the victim experienced less suffering (i.e., less emotional trauma and physical pain).

### 4.1. Economic Deprivation and Prejudice

The findings support and extend the motivated perception perspective, which identifies ways in which economic deprivation can motivate majority group members to engage in perceptual processes that disadvantage minority group members and increase the likelihood of discrimination [13,17]. The findings also provide a number of important extensions to extant economic deprivation and prejudice literature. First, we demonstrated that in two countries, economic hardship can alter perceptions of the emotional and physical suffering of a minority victim of violent assault. Second, we showed that altered perceptions of suffering may have consequential implications for legal outcomes in violent assault cases. Specifically, the perception that the victim experienced less suffering predicted reduced support to compensate the victim or to punish the assailant. Thus, these findings provide novel evidence of ways in which motivated perceptual bias may facilitate a social climate that is more permissive of violence toward minorities.

### 4.2. Implications for Public Health and Intergroup Relations

The findings have significant implications for public health and intergroup relations. Tacit support for violence directed at minorities can occur when people perceive less prejudice in perpetrators of “hate crimes.” Feelings of disempowerment can serve as a central mechanism for tacit support [42]. Importantly, the findings reveal another method of implicit or unspoken support for violence: diminished recognition of emotional trauma and physical pain experienced by the victim. Specifically, difficult life circumstances such as economic hardship were positively associated with less recognition of suffering when the victim was Chinese. Furthermore, the findings indicate that additional harm to the victim and society can result from tacit support, as shown through reduced support to compensate the victim and reduced likelihood to support criminal charges against the assailant (which may increase the likelihood of future criminal action by the assailant).

Societal reactions to discrimination and violence can impact the mental health of individuals who are victimized and others. There is growing evidence that various forms of support provided by others, including a sensitivity to suffering, may help buffer negative mental health outcomes [43,44]. For example, Asians who experienced COVID-19-related discrimination reported fewer negative mental health outcomes when others provided emotional and social support [45]. Further, people may report less pain when others empathize with their suffering [46,47]. Research is needed to examine these mental health and public health issues in greater detail in the context of the pandemic.

### 4.3. Limitations and Future Research

There are a number of limitations to the current study. The sample, which was recruited using the crowdsourcing platform MTurk, may be less representative than national probability samples. As a result, the findings may not generalize to all persons in the United States or India. We were not able to control the environment in which participants completed the measures [48]. However, the selection process required participants to have a strong MTurk reputation score that is typically sufficient to ensure a good quality participant pool [33], and the participants successfully answered numerous attention checks. The vignettes describing the incident were presented in written form, and it is possible that stronger responses might have resulted if the scenarios were presented in a more vivid manner such as through a video or interactive computer simulation. The victim and assailant in the scenario were male. Research is needed to examine in more detail how gender and the race of the those involved may influence responses. As the majority of participants had some level of college education, research is needed to explore how other individual difference variables are associated with reactions to violence. Finally, demand effects are a concern in any social psychological experiment, especially those involving race-related matters. However, the consistent two-way interactions in both the United States and India diminish concerns regarding demand bias because participants in both countries would have to know to adjust their reactions to the experimental manipulation as a function of their item endorsements on the economic hardship measures.

According to the U.S. Federal Bureau of Investigations Hate Crime Statistics Report [49], in 2019 there were a record number of hate crime murders, greater than twice the number the year prior. In 2020, hate crimes against Asians increased nearly 150% in the United States [50]. These startling increases raise pressing issues for future research. Research should explore whether the current findings are limited to COVID-19-related assaults or whether they will generalize to other forms of violence against disadvantaged group members. It would also be appropriate to examine whether the current findings would generalize to violence against minorities that is oftentimes not perceived as being associated with a hate crime, such as police violence against Black men and women. This is relevant because recent analyses of police shootings in the United States indicate that Black unarmed males are seven times more likely than White males to be killed by police gunfire [51]. Clearly, these questions are highly relevant to public health, jury selection, and decision-making processes. Finally, economic downturns are associated with an increase in intimate partner violence [52,53,54,55]. An examination of how coping with catastrophic events such as natural disasters [56] and wars may influence reactions to intimate partner violence or minority group mistreatment is warranted.

## 5. Conclusions

In the present study, a completely innocent victim suffered a barrage of disparaging remarks, endured three kicks to the chest, was knocked to the floor, and faced further threats while on the floor. Despite the brutality of this unjustified attack, COVID-19-driven economic hardship experienced by participants predicted an array of reactions that reflected reductions in the recognition of the victim’s civil and human rights when the victim was Chinese. High degrees of economic hardship predicted diminished certainty that the victim suffered emotional trauma and physical pain, lowered support to compensate the victim for his suffering and weakened support to criminally charge the assailant.

The findings underscore the pressing need for prompt action to mitigate economic hardship and to address racism and discrimination. The World Health Organization, American Public Health Organization, American Psychological Association, other public health entities, and leaders worldwide have called for swift measures to prevent discrimination, stigma, and violence as a result of the COVID-19 pandemic [9,57,58]. First and foremost, factual information concerning the virus, including the manner in which it is spread, the symptoms and health issues that can develop if exposed to the virus, and specific and effective actions that reduce or prevent exposure are essential. Testing and treatment options should be clearly provided with no to minimal barriers to access services. Information should plainly discuss the negative consequences for the medical and public health system and public health in general if hospitals and medical providers are overwhelmed with too many patients. In addition, educational campaigns designed to reduce stigma and discrimination and violence should underscore fundamental principles of basic human rights, our shared humanity, and empathy for all individuals [56,58].

## Figures and Tables

**Table 1:** Correlations between the major variables of interest.

Variable	1	2	3	4	5
**Chinese Victim**					
1. Participant economic hardship	-				
2. Perceived victim emotional trauma	−0.17 *	-			
3. Perceived victim pain	−0.22 *	0.39 ***	-		
4. Victim compensation support	−0.17 *	0.33 ***	0.25 ***	-	
5. Criminal charges endorsement	−0.16 *	0.33 ***	0.37 ***	0.43 ***	-
**White Victim**					
1. Participant economic hardship	-				
2. Perceived victim emotional trauma	0.04	-			
3. Perceived victim pain	−0.01	0.17 *	-		
4. Victim compensation support	0.12	0.31 ***	0.22 **	-	
5. Criminal charges endorsement	0.04	0.28 ***	0.24 ***	0.36 ***	-

* *p* < 0.05. ** *p* < 0.01. *** *p* < 0.001 (2-tailed tests of significance).

## Data Availability

The data presented in this study are available on request from the corresponding author.
